# Regional common prosperity level and its spatial relationship with carbon emission intensity in China

**DOI:** 10.1038/s41598-023-44408-9

**Published:** 2023-10-09

**Authors:** Xiaochun Zhao, Laichun Long, Shi Yin

**Affiliations:** 1 School of Management, Anhui University, Hefei, 230601 China; 2 College of Economics and Management, Hebei Agricultural University, Baoding, 071001 China

**Keywords:** Environmental economics, Environmental sciences, Environmental social sciences

## Abstract

The characteristics of common prosperity include harmonious relationships between humans and the environment, as well as sustainable economic and social growth. The process of achieving common prosperity will necessarily have an impact on carbon emissions. In this article, panel statistics collected from 30 Chinese provinces and cities between the years 2006 and 2020 are utilized to assess the level of common prosperity and the intensity of carbon emissions in China. Then the SDM model is applied to explore the effects of the common prosperity level on the intensity of carbon emissions. The findings reveal that: (i) The common prosperity level in China has shown an increasing tendency. Between 2006 and 2020, the mean level of common prosperity increased from 0.254 to 0.486. From the regional perspective, eastern China has seen greater levels of common prosperity than central China, while central China has experienced greater levels of common prosperity than western China; regional disparities in the degree of common prosperity are substantial among Chinese provinces from 2006 to 2020; the common prosperity level is relatively high in economically developed provinces and relatively low in economically backward provinces. (ii) China's carbon emission intensity shows a continuous downward tendency. The annual average intensity of China's carbon emissions decreased from 4.458 in 2006 to 2.234 in 2020. From the regional perspective, the three main regions' carbon emission intensity likewise exhibits a decline in tendency between 2006 and 2020; still, western China continues to have the greatest carbon emission intensity, following central China, while eastern China has the smallest; however, certain provinces, notably Inner Mongolia and Shanxi, continue to have high carbon emission intensity. (iii) China's common prosperity level and carbon emission intensity both exhibit positive spatial autocorrelation at a 1% significant level under the adjacency matrix. The spatial agglomeration effect is significant, and adjacent provinces can affect each other. (iv) The SDM (Spatial Durbin Model) model test with fixed effects finds that the increase in the level of common prosperity suppresses the intensity of carbon emissions in the local area and neighboring regions. (v) The mediating effects model indicates that the process of common prosperity suppresses carbon emission intensity through high-quality economic development, narrowing the income disparity, and the development of a sharing economy.

## Introduction

The threat of global warming has emerged as a major worldwide concern. The United Nations 2030 Agenda for Sustainable Development unequivocally states that immediate action is required to cut carbon emissions in order to address climate change^1^. Since 1978, China's rapid development has consumed vast amounts of fossil energy, significantly increasing carbon emissions. In 2020, China emitted 98.935 million metric tons of CO_2_ into the atmosphere, which was equivalent to 30.93% of world emissions. The Chinese government has committed to reaching carbon peaking by 2030 and carbon neutrality by 2060, indicating that reducing the tremendous growth in carbon emissions is a current priority. At the same time, the Chinese government is solemnly promoting the objectives of reducing income inequality, enhancing people's livelihoods, and achieving common prosperity for all. Common prosperity is a concept of social development based on material prosperity and the gradual improvement of people's living standards. Its core objective is to enable the wealth and resources of society as a whole to be distributed fairly and reasonably to all its members, to narrow the gap between the rich and the poor, and to enable all the people to share in the fruits of economic growth and social progress, ultimately realizing both material and spiritual prosperity^2–4^. China advocates a high-quality common prosperity that includes material, spiritual, and ecological civilization, which imposes stricter requirements for strengthening environmental protection in economic development. Historical experience shows that material wealth represented by GDP is not the only measure of affluence. The development model at the expense of the ecological environment can only be exchanged for short-term economic growth^4^. The scientific connotation of common prosperity includes harmony between human beings and nature. China's pursuit of high-quality common prosperity is bound to put forward energy-saving and carbon-reduction work requirements. Therefore, reducing CO_2_ emission intensity is not only an essential means for China to achieve the "double carbon goal" on schedule but also an essential requirement for achieving common prosperity. However, the academic community has not yet clarified how common prosperity affects carbon emissions. Analyzing the effect of common prosperity on carbon emission intensity can serve as a guide for scientifically formulating carbon emission reduction planning in the process of realizing common prosperity. This has important practical implications for hastening to achieve the "double carbon goal" and supporting high-quality Chinese development.

The rest of this article can be divided into the following sections: First, the relevant study findings on common prosperity and carbon emissions are reviewed. Then, it introduces the research methodology and data sources utilized in the article. Next, the study's findings and an analysis of how the common prosperity level affects the intensity of carbon emissions are presented. Finally, according to the study results, the article provides a relevant discussion. On the basis of the findings, relevant suggestions are made, and this paper's shortcomings and future prospects are outlined.

## The influence mechanism of common prosperity on carbon emission intensity

**Figure 1:**
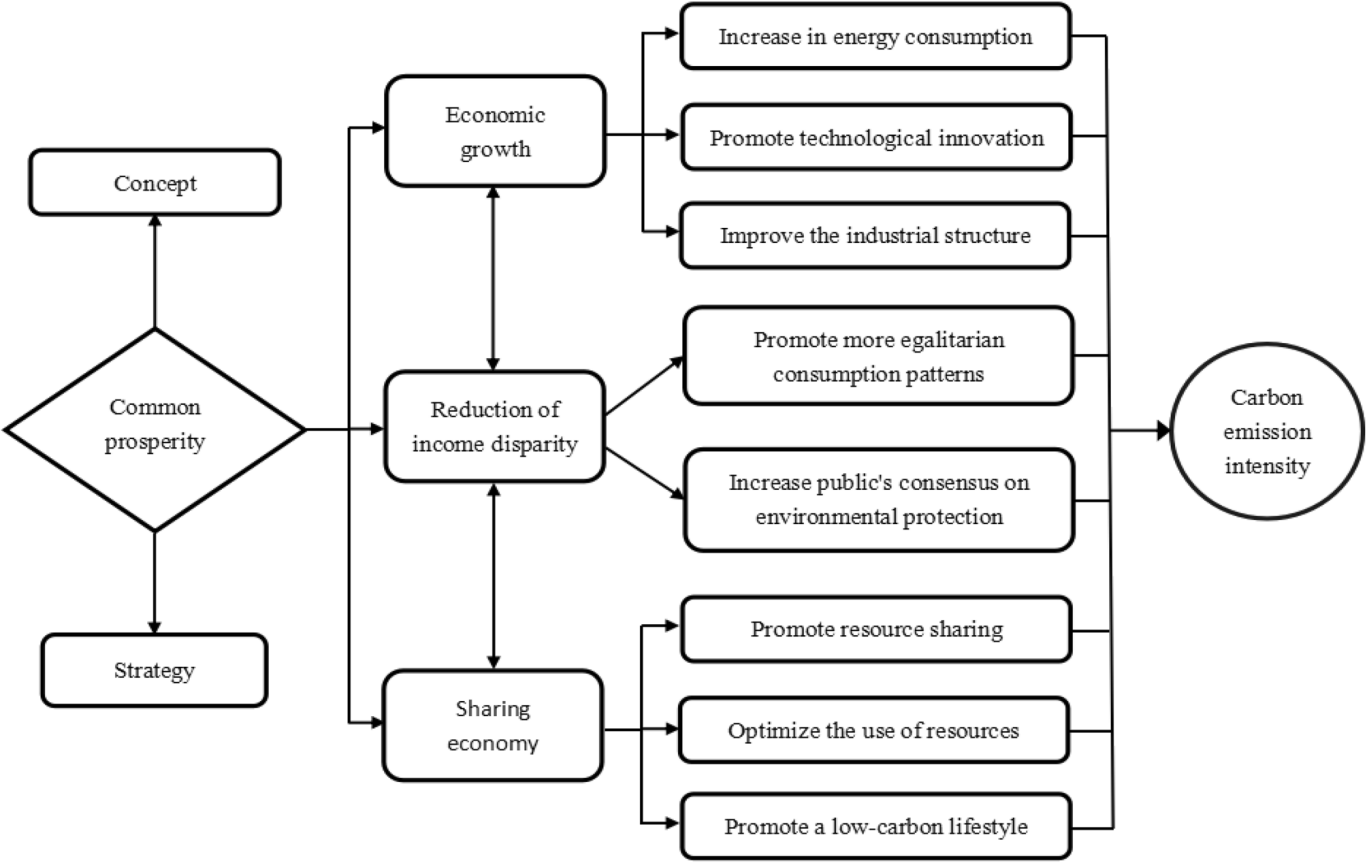
The influence mechanism of common prosperity on carbon emission intensity.

The influence of common prosperity on CO_2_ emissions has yet to be extensively studied in the scholarly literature. In a society that is experiencing common prosperity, the society's overall wealth rises, the income gap continues to close, and everyone benefits from economic growth. Its realization is a systematic project that inevitably impacts carbon emissions. Therefore, this study starts from the connotation and critical paths of common prosperity to sort out the mechanism of common prosperity's influence on carbon emission intensity. See Fig. 1 for details.

(1) The concept and realization of common prosperity

Firstly, the connotation of common prosperity can be summarized in three dimensions. ① Common prosperity means eliminating absolute poverty and gradually reaching material affluence^5^. ② Common prosperity belongs to all people, and people who participate in achieving common prosperity can share the wealth^6^. ③ Common prosperity includes political, economic, cultural, social, and ecological "prosperity" in all aspects. And common prosperity should include not just economic accomplishment but also mental as well as environmental prosperity for the people^7^.

Secondly, the characteristics of common prosperity have three fundamental characteristics. They are development, sharing, and sustainability, respectively. ① Development means the increase of the total wealth of society and the rise in the income level of the people, which is the basis for the realization of common prosperity^8^. ② Sharing indicates that the material wealth created should benefit everyone with equal employment opportunities, health care, education, and the ecological environment^9^. ③ Sustainability means not only regulating income distribution patterns to achieve coordinated growth in cities and villages as well as different areas, but also achieving intergenerational equity with future generations through reducing CO_2_ emissions and conserving energy. It is necessary to achieve common prosperity for the present generation and create the necessary material conditions for future generations to achieve prosperity^10^.

Finally, the realization of the strategy of common prosperity relies on three critical paths: vigorous economic development, continuous narrowing of the gap between the rich and the poor, and ensuring that people share the fruits of development^11^. ① Economic development: The core of the common prosperity strategy is to promote sustained economic growth. By fostering economic expansion and creating more employment opportunities and wealth accumulation, a solid foundation can be laid for achieving common prosperity. The government and enterprises need to increase support for industries, encourage innovation and entrepreneurship, and enhance productivity and output, thus providing more opportunities and benefits for people^12^. ② Reducing the gap between the rich and the poor: In implementing the strategy of common prosperity, it is important to focus on narrowing the wealth disparity to ensure a more equitable distribution of resources. The government can achieve this through tax policies, social welfare programs, and poverty alleviation measures^13^. ③ Sharing the benefits of development: Shared prosperity emphasizes allowing all members of society to partake in the benefits of development. Governments need to strengthen the provision of basic public services, such as healthcare, education, and housing. Additionally, they should encourage public involvement in social affairs and enhance social cohesion^14^.

(2) Common prosperity affects carbon emission intensity through economic growth

The basis for common prosperity is economic growth. A good economy provides greater opportunities and resources for society as a whole. Economic development provides the basis for job creation, higher productivity and income levels, and improved infrastructure and public services in society^15^. However, economic development is usually accompanied by an increase in energy consumption, in particular, the use of fossil fuels, such as coal, oil, and natural gas. The widespread use of these fossil fuels in energy production, transportation, and industrial production has led to an increase in carbon dioxide emissions^16^. As the economy continues to develop, people are beginning to realize the impact of carbon emissions on the environment and climate. In order to achieve sustainable development, governments and enterprises are gradually realizing the importance of reducing carbon emissions. As a result, more resources are being invested to promote scientific and technological innovation and technological upgrading to reduce energy consumption and carbon emissions. The decoupling of economic growth from carbon emissions is achieved through the introduction of cleaner and more efficient energy technologies, as well as the improvement of production and transportation processes^17^. Additionally, the government will introduce a series of environmental policies and regulations. These policies and regulations will incentivize enterprises to adopt more environmentally friendly production methods and encourage the industrial structure to develop in a low-carbon direction. The environmental protection industry is gradually becoming a hot spot for investment and market demand^18^. Investors and consumers are increasingly concerned about environmentally friendly and low-carbon products, which also prompts companies to shift to more environmentally friendly products, thus promoting the upgrading of the industrial structure. And the transition to more environmentally friendly and low-carbon industries can further reduce carbon emissions^19^.

(3) Common prosperity affects carbon emission intensity by reducing the income disparity

The key to common prosperity is the continuous narrowing of the income gap. Common prosperity seeks a fair distribution of wealth in society as a whole, and narrowing the real income gap is an important way to realize common prosperity. Reducing income disparity has a positive impact on reducing carbon emission intensity. On the one hand, larger income gaps usually lead to high consumption and waste among the affluent. In contrast, poorer people may not be able to afford greener choices. Therefore, narrowing the income gap can promote more egalitarian consumption patterns and reduce carbon-emitting lifestyles, thereby lowering the overall intensity of carbon emission intensity^20^. On the other hand, by narrowing the income gap, it can increase the public's consensus on protecting the environment. This will help the government to better formulate low-carbon environmental protection policies and enhance the effectiveness of the implementation of low-carbon policies^21^. A smaller income gap will increase the importance that low-income people attach to environmental issues, and thus more actively support and participate in activities and policies to reduce carbon emissions^22^. In addition, governments are more likely to adopt more balanced environmental policies when income disparity is small, which is conducive to reducing overall carbon emissions^23^.

(4) Common prosperity affects carbon emission intensity through the sharing economy

Vigorously developing the sharing economy is an inevitable requirement for the construction of common prosperity. Common prosperity emphasizes sharing the fruits of economic growth and social progress among more people, not just a few or a specific group. Through the development of the sharing economy, employment opportunities can be expanded, innovation and entrepreneurship can be promoted, and sustainable social development can be fostered^24^. The development of the sharing economy has a positive impact on reducing carbon emission intensity. The sharing economy is usually centred on resource sharing and optimal utilization, which can effectively reduce the waste of resources and energy^25^. In addition, the sharing economy encourages people to use resources in a more rational and environmentally friendly manner, which can promote a low-carbon lifestyle^26^. By creating a sharing economy, the public infrastructure will be improved, and individuals may significantly reduce their carbon footprints through a shared lifestyle^27^.

To summarize, common prosperity as a whole has a dampening effect on carbon emission intensity. In the process of realizing common prosperity, narrowing the income gap and developing a sharing economy can reduce the intensity of carbon emissions. In the short term, economic growth will increase carbon emissions, but with technological progress and increased awareness of environmental protection, carbon emissions can be reduced in the future through a sustainable development path.

Combining the literature, it is possible to find an abundance of research on the various factors that affect carbon emissions. Still, there are few studies on the direct impact of common prosperity on carbon emissions. This paper's marginal contribution and innovation point exist: It helps enrich the research content about CO_2_ emission influencing factors and provides an important reference and scientific basis for formulating carbon emission reduction policies. Additionally, this study develops a common prosperity indicator system based on three perspectives: sustainability, sharing, and development, which can more accurately and comprehensively assess the degree of common prosperity. Achieving common prosperity is not only about creating material wealth and reducing the wealth and poverty disparity. It is also about strengthening ecological civilization and achieving harmony between human beings and nature. China's pursuit of high-quality common prosperity necessarily requires accelerating energy-saving and carbon-reduction efforts. Therefore, this study evaluates the common prosperity level and the intensity of carbon emissions in China using data from Chinese provinces between 2006 and 2020, then applies the SDM model to explore how common prosperity impacts the intensity of carbon emissions, which inspires the formulation of scientific CO_2_ dioxide emissions reduction policies in the process of achieving common prosperity in China.

## Research design

### Common prosperity level measurement

#### Selection of measurement indicators

A system of indicators needs to be designed to measure common prosperity levels. This study is based on wholeness, science, and data availability principles. Combined with the previous summary analysis of the characteristics of common prosperity and referring to the relevant findings of previous scholars, this paper designs the common prosperity indicator system in three dimensions: sustainability, sharing, and development. Twelve tertiary indicators, such as per capita disposable income of residents (yuan/person), per capita consumption expenditure of residents (yuan/person), the Gini coefficient, the proportion of people in higher education (%), research and development (R&D) investment intensity (%), forest coverage (%), GDP per capita (yuan/person), and so on, were selected^28–30^. Detailed, specific indicators can be seen in Table 1.

**Table 1:** Indicators system of common prosperity.

First-level indicators	The secondary indicators	Three-level indicator (Unit)	Nature of indicators
Development	Affluence	Per capita disposable income of residents (yuan/person)	Positive
		Engel coefficient (%)	Negative
		Per capita consumption expenditure of residents (yuan/person)	Positive
	Common	Gini coefficient	Negative
		Multiplier difference between urban and rural residents' income	Negative
		Tyre Index	Negative
Sharing	Cultural education	Proportion of people in higher education (%)	Positive
	Medical health	Number of beds per 1,000 people in medical institutions (pcs)	Positive
	Infrastructure	Public transportation vehicles per 10,000 people (units)	Positive
Sustainability	Technology innovation	R&D investment intensity (%)	Positive
	Ecological environment	Forest coverage (%)	Positive
	Development quality	GDP per capita (yuan/person)	Positive

#### Measurement method

The common prosperity level is evaluated using the entropy value approach. It efficiently avoids human-caused bias and is frequently used in evaluating comprehensive levels^31^. And the computation equation is (1)–(5).

In the first step, the primary data must be normalized to remove the effects of the varied dimensions and each indicator's units. The formula for this step is as follows:

Positive:

1 $${m}_{ij}=\frac{{n}_{ij}-\mathrm{min}\left({n}_{ij}\right)}{\mathrm{max}\left({n}_{ij}\right)-\mathrm{min}({n}_{ij})}$$

Negative:

2 $${m}_{ij}=\frac{\mathrm{max}\left({n}_{ij}\right)-{n}_{ij}}{\mathrm{max}({n}_{ij})-\mathrm{min}({n}_{ij})}$$

where ${ n}_{ij }$ denotes indicator data, $\mathrm{max}({n}_{ij})$ and $\mathrm{min}({n}_{ij})$ indicate the highest and lowest values in the data, and ${ m}_{ij}$ is the value that has been processed.

The second step is to measure the relative weights of every indicator. The formula for this step is as follows:

3 $${z}_{ij}=\frac{{m}_{ij}}{\sum_{i=1}^{x}{y}_{ij}}; {o}_{j}=\left[-\frac{1}{\mathrm{ln}\left(x\right)}\right]\sum_{i=1}^{x}{z}_{ij}{\mathrm{ln}(z}_{ij})$$

where ${z}_{ij}$ is the indicator's weight, and ${o}_{j}$ denotes the information entropy of the indicator.

4 $${y}_{j}=(1-{o}_{j})/\sum_{j=1}^{x}\left(1-{o}_{j}\right)$$

where ${y}_{j}$ denotes the entropy value, and $1-{o}_{j}$ is the information utility value.

In the last step, use the following equation to evaluate the common prosperity level (*CP*):

5 $$CP=\sum_{j=1}^{x}{y}_{j}{.m}_{ij}; \sum_{j=1}^{x}{y}_{ij}=1$$

## The intensity of carbon emission measurement

CO_2_ emissions per 10,000 yuan of GDP are used to measure the intensity of carbon emissions, which is more scientific than using carbon dioxide emissions directly^32^, as shown in Eq. (6).

6 $$ CI = C_{i} /GDP $$

In Eq. (6), *CI* is the intensity of CO_2_ emissions; *C*_*i*_ denotes the CO_2_ dioxide emissions of the consumed energy.

Referring to the latest research, this article chooses the CO_2_ emissions produced by eight different energy sources as the total carbon dioxide emissions from energy consumption^33^. As shown in Eq. (7).

7 $$ C_{i} = \sum\limits_{i = 1}^{8} {E_{i} \times SCC_{i} } \times CEF_{i} $$

where *i* denotes the type of energy consumption in each area; *E*_*i*_ denotes the total CO_2_ dioxide emissions; *SCC*_*i*_ is the discount factor of energy; and *CEF*_*i*_ denotes the CO_2_ dioxide emissions factor.

### Model selection

Unlike traditional econometric models (Examples: Linear Regression Model, Logistic Regression Model, Time Series Model, Panel Data Model, etc.), spatial econometric models consider the spatial dependence and spillover effects among regions. Spatial econometric modeling is chosen because the research topic of this paper involves the influence of geospatial factors. Spatial econometric models are able to take into account geospatial interdependencies, and they are more applicable than traditional econometric models when dealing with spatially correlated data. The research question in this paper needs to consider the possible spatial correlations and interactions between different regions. Therefore, the use of spatial econometric models can more accurately analyze the direct effects and indirect spillover effects of common prosperity on carbon emission intensity^34^. And spatial correlation of data is a prerequisite for using spatial econometric models, so spatial correlation tests must be conducted for common prosperity level and carbon emission intensity, respectively.

#### Spatial correlation examination

1. Global Spatial Correlation Examination

The global spatial correlation can test the overall characteristics of the common prosperity level and carbon emission intensity, measured by the global Moran index (Moran’s *I*). It takes values in the range of [− 1, 1]. When *I* is positive (negative), it represents that the spatial correlation of the common prosperity level or the intensity of carbon emissions in each province in China is positive (negative). The calculation method is shown in Eq. (8).

8 $$ I = \frac{{n\sum\limits_{i = 1}^{n} {\sum\limits_{j = 1}^{n} {W_{ij} \left( {Z_{i} - \overline{Z} } \right)\left( {Z_{j} - \overline{Z} } \right)} } }}{{\sum\limits_{i = 1}^{n} {\sum\limits_{j = 1}^{n} {W_{ij} \sum\limits_{i = 1}^{n} {\left( {Z_{i} - \overline{Z} } \right)^{2} } } } }} $$

where *n* denotes each province; *Z* represents the common prosperity level or carbon emission intensity; and *W* indicates the spatial weight matrix. Referring to the study by Chen et al., three matrices of adjacency matrix (*W*_1_), geographic distance matrix (*W*_2_), and economic distance matrix (*W*_3_) are constructed to test the global spatial autocorrelation of the common prosperity level and the intensity of carbon emissions, respectively. After considering the results of the examinations, the most appropriate spatial weight matrix is then chosen^35^. See Eqs. (9), (10), and (11) for details.

① Adjacency matrix (*W*_*1*_)
  9 $$ W_{ij} = \left\{ \begin{gathered} {1}\quad {\text{(If}}\;{\text{region}}\;i\;{\text{and}}\;{\text{region}}\;j\;{\text{are}}\;{\text{geographically}}\;{\text{contiguous)}} \hfill \\ {0}\quad {\text{(If}}\;{\text{region}}\;i\;{\text{and}}\;{\text{region}}\;j\;{\text{are}}\;{\text{not}}\;{\text{geographically}}\;{\text{contiguous)}} \hfill \\ \end{gathered} \right. $$
② Geographic distance matrix (*W*_*2*_)
  10 $$ W_{ij} = \frac{1}{{d_{ij} }}(d_{ij} \;{\text{is}}\;{\text{the}}\;{\text{straight - line}}\;{\text{distance}}\;{\text{between}}\;{\text{provincial}}\;{\text{capitals}}) $$
③ Economic distance matrix (*W*_*3*_)
  11 $$ W_{ij} = \frac{1}{{\left| {\left. {\overline{Y}_{i} - \overline{Y}_{j} } \right|} \right.}}(\overline{Y}_{i} \;{\text{and}}\;\overline{Y}_{j} \;{\text{are}}\;{\text{the}}\;{\text{GDP}}\;{\text{per}}\;{\text{capita}}\;{\text{of}}\;{\text{region}}\;i\;{\text{and}}\;{\text{region}}\;j{,}\;{\text{respectively}}) $$

(2) Local spatial correlation examination

Each region's spatial clustering and dispersion characteristics could be analyzed in more depth using the local Moran index. Stata software is used to construct the local Moran diagram, which can visualize the local spatial correlation of the common prosperity level and the intensity of carbon emissions in each area. The calculation formula is shown in (12).

12 $$ I_{i} = \frac{{(Z_{i} - \overline{Z})}}{{\sum\limits_{i = 1}^{n} {(Z_{i} - \overline{Z})^{2} } }}\sum\limits_{j = 1 \cdot j \ne i}^{n} {W_{ij} (Z_{j} - \overline{Z})} $$

where *I* represents the respective Moran values of different provinces.

#### Spatial Durbin model

Finally, it is determined that a spatial econometric model could be used, and a suitable spatial matrix is selected. Model testing selects the SDM model to examine the influence of the level of common prosperity on the intensity of CO_2_ emissions. Its calculation formula is shown in (13). *β* denotes the explanatory variable's coefficient; *ε* represents the random error; *i* represents each province; *t* is the time; *ρ* denotes the coefficient of spatial correlation. To remove the influence of heteroskedasticity, this article log-transforms all variables to ensure smooth data^36^.

13 $$ \begin{gathered} \ln CI_{{{\text{it}}}} = \beta_{0} + \beta_{1} \ln CP_{{{\text{it}}}} + \beta_{2} \ln IS_{{{\text{it}}}} + \beta_{3} \ln UR_{{{\text{it}}}} + \beta_{4} \ln TI_{{{\text{it}}}} + \beta_{5} \ln OU_{{{\text{it}}}} + \beta_{6} \ln FD_{{{\text{it}}}} + \rho \ln CI_{{{\text{it}}}} \hfill \\ \quad \quad \quad + \theta_{1} W\ln CP_{{{\text{it}}}} + \theta_{2} W\ln IS_{{{\text{it}}}} + \theta_{3} W\ln UR_{{{\text{it}}}} + \theta_{4} W\ln TI_{{{\text{it}}}} + \theta_{5} W\ln OU_{{{\text{it}}}} + \theta_{6} W\ln FD_{{{\text{it}}}} \varepsilon_{{{\text{it}}}} \hfill \\ \varepsilon_{{{\text{it}}}} = \rho W\varepsilon_{{\text{i}}} + U\varepsilon_{{\text{i}}} ,U\varepsilon_{{\text{i}}} \sim {\text{ii}}d\left( {0,\sigma^{2} } \right) \hfill \\ \end{gathered} $$

In Eq. (13), ln*CI* is the explained variable, and *CI* is the intensity of CO_2_ emissions. ln*CP* denotes the core explanatory variable, and *CP* is the common prosperity level. To prevent omitting important control variables that may bring about endogeneity problems. This paper refers to existing studies to sort out essential control variables that impact carbon emissions and selects industrial structure, urbanization, technological innovation, opening to the outside world, and financial development as controlling factors^37–39^. Among them, the logarithm of the ratio of the output of secondary industry to GDP is used to measure industrial structure (ln*IS*); urbanization (ln*UR*) is expressed as the logarithm of the ratio of urban residents to all people; the logarithm of the number of domestic patent applications issued is used to measure technological innovation (ln*TI*); opening to the outside world (ln*OU*) is represented by the logarithm of the proportion of total imports and exports to GDP; financial development (ln*FD*) is expressed as the logarithm of the amount of deposits and loans made by banking and financial institutions as a percentage of total GDP.

#### Mediation effects model

In order to test the mechanism of common prosperity on carbon emission intensity, this paper constructs a mediation effect model. Mediating effects modeling helps to explain the mechanism behind the relationship between two variables. It elucidates why and how one variable influences the other. By identifying the mediating variable, researchers can gain a deeper understanding of the relationship^40^. The calculation formula is shown in (14).

14 $$ \begin{gathered} \ln H_{{\text{i,t}}} = \beta_{0} + \beta_{1} \ln H_{{\text{i,t - 1}}} + \beta_{2} \ln CP_{{{\text{it}}}} + \sum {\beta_{K} \ln X_{{{\text{it}}}} } + \varepsilon_{{{\text{it}}}} \hfill \\ \ln CI_{{\text{i,t}}} = \delta_{0} + \delta_{1} \ln CI_{{\text{i,t - 1}}} + \delta_{2} \ln CP_{{{\text{it}}}} + \delta_{3} \ln H_{{\text{i,t}}} + \sum {\beta_{K} \ln X_{{{\text{it}}}} } + \varepsilon_{{{\text{it}}}} \hfill \\ \end{gathered} $$

In Eq. (14), ln*H* represents the mediating variable, which is indicated by economic growth (ln*EG*), the income disparity (ln*ID*), and sharing economy (ln*SE*), respectively. And economic growth is expressed in terms of gross domestic product per capita; income disparity is expressed in terms of the Gini coefficient; and the sharing economy is expressed in terms of the volume of transactions in the sharing economy market. If the coefficients *β*_1_ and*δ*_3_, ln*H* are significant, ln*H* acts as a mediator in the relationship between common prosperity-carbon emission intensity.

### Sources of information and descriptive statistics

In this article, data from 30 Chinese provinces and cities (2006–2020) are chosen for empirical research considering the data's accessibility (Taiwan, Macao, Tibet, and Hong Kong are missing a lot of data and are not taken into account). The indicator system's data is derived from officially published information from the national statistics bureau and the province's official report. The results of the data description statistics can be seen in Table 2.

**Table 2:** Variable descriptive statistics.

Variables	Number	Standard deviation	Minimum value	Average value	Maximum value
ln*CI*	450	0.682	− 1.137	0.779	2.558
ln*CP*	450	0.333	− 2.110	− 1.038	− 0.263
ln*IS*	450	0.232	− 1.845	− 0.825	− 0.486
ln*UR*	450	0.241	− 1.292	− 0.622	− 0.110
ln*TI*	450	1.619	4.575	9.585	13.473
ln*OU*	450	0.972	− 4.943	− 1.742	0.512
ln*FD*	450	0.331	0.253	1.039	2.096
ln*EG*	450	0.572	− 1.427	0.689	1.878
ln*ID*	450	450	0.342	− 2.025	− 0.724
ln*SE*	450	450	0.672	− 3.343	− 1.872

## Common prosperity level and carbon emission intensity evaluation results

### Common prosperity level evaluation

The common prosperity level in different Chinese provinces between 2006 and 2020 could be assessed by using the entropy value method. The 30 provinces are then categorized according to where they are located on the country’s map: eastern China, central China, and western China. Table 3 displays the results.

**Table 3:** Common prosperity level in China from 2006 to 2020.

Region	Province	2006	2007	2008	2009	2010	2011	2012	2013	2014	2015	2016	2017	2018	2019	2020
Eastern China	Beijing	0.472	0.529	0.525	0.553	0.583	0.594	0.580	0.593	0.641	0.669	0.657	0.732	0.730	0.737	0.769
	Tianjin	0.324	0.337	0.394	0.423	0.407	0.461	0.485	0.482	0.532	0.493	0.519	0.538	0.558	0.554	0.522
	Hebei	0.249	0.260	0.265	0.273	0.257	0.297	0.356	0.373	0.387	0.391	0.396	0.446	0.441	0.449	0.493
	Liaoning	0.325	0.274	0.290	0.360	0.354	0.367	0.365	0.378	0.413	0.482	0.467	0.506	0.495	0.514	0.475
	Shanghai	0.400	0.395	0.447	0.464	0.462	0.533	0.508	0.523	0.490	0.559	0.615	0.653	0.621	0.618	0.667
	Jiangsu	0.291	0.334	0.264	0.291	0.326	0.412	0.371	0.430	0.433	0.463	0.464	0.552	0.555	0.572	0.520
	Zhejiang	0.406	0.407	0.407	0.428	0.417	0.485	0.446	0.520	0.571	0.558	0.550	0.561	0.611	0.628	0.587
	Fujian	0.283	0.349	0.293	0.344	0.378	0.367	0.366	0.404	0.444	0.508	0.462	0.525	0.513	0.560	0.581
	Shandong	0.291	0.286	0.284	0.319	0.339	0.338	0.322	0.404	0.421	0.451	0.485	0.489	0.468	0.517	0.447
	Guangdong	0.246	0.323	0.292	0.346	0.333	0.379	0.401	0.381	0.428	0.496	0.515	0.488	0.504	0.504	0.491
	Hainan	0.270	0.227	0.216	0.234	0.272	0.318	0.293	0.343	0.400	0.376	0.389	0.428	0.465	0.480	0.485
	Mean	0.323	0.338	0.334	0.367	0.375	0.414	0.409	0.439	0.469	0.495	0.502	0.538	0.542	0.557	0.549
Central China	Shanxi	0.193	0.256	0.214	0.293	0.297	0.262	0.265	0.333	0.311	0.317	0.342	0.400	0.406	0.443	0.410
	Jilin	0.322	0.299	0.289	0.339	0.367	0.385	0.337	0.368	0.426	0.433	0.443	0.429	0.424	0.476	0.490
	Heilongjiang	0.321	0.292	0.301	0.311	0.358	0.396	0.401	0.410	0.470	0.475	0.469	0.468	0.475	0.525	0.473
	Anhui	0.157	0.226	0.235	0.217	0.285	0.238	0.308	0.284	0.390	0.354	0.378	0.375	0.401	0.410	0.441
	Jiangxi	0.239	0.276	0.295	0.320	0.336	0.310	0.361	0.381	0.410	0.416	0.423	0.480	0.445	0.430	0.455
	Henan	0.197	0.261	0.260	0.221	0.263	0.277	0.302	0.288	0.340	0.405	0.360	0.423	0.399	0.414	0.495
	Hubei	0.273	0.272	0.243	0.280	0.304	0.285	0.364	0.403	0.391	0.403	0.424	0.474	0.496	0.533	0.510
	Hunan	0.284	0.231	0.260	0.274	0.286	0.364	0.377	0.340	0.438	0.469	0.465	0.447	0.481	0.491	0.504
	Mean	0.248	0.264	0.262	0.282	0.312	0.315	0.339	0.351	0.397	0.409	0.413	0.437	0.441	0.465	0.472
Western China	Inner Mongolia	0.225	0.234	0.275	0.270	0.260	0.336	0.305	0.336	0.355	0.348	0.382	0.382	0.425	0.423	0.466
	Guangxi	0.190	0.229	0.167	0.231	0.230	0.241	0.304	0.283	0.359	0.398	0.364	0.403	0.423	0.435	0.427
	Chongqing	0.174	0.209	0.226	0.286	0.267	0.350	0.321	0.370	0.385	0.400	0.411	0.469	0.490	0.499	0.513
	Sichuan	0.241	0.204	0.263	0.233	0.258	0.331	0.293	0.390	0.361	0.414	0.407	0.469	0.457	0.448	0.489
	Guizhou	0.139	0.121	0.170	0.125	0.153	0.181	0.201	0.242	0.337	0.335	0.383	0.370	0.433	0.402	0.360
	Yunnan	0.138	0.169	0.128	0.180	0.257	0.218	0.250	0.290	0.326	0.330	0.347	0.407	0.444	0.431	0.451
	Shaanxi	0.201	0.231	0.258	0.283	0.304	0.327	0.339	0.379	0.393	0.451	0.461	0.440	0.470	0.468	0.570
	Gansu	0.125	0.132	0.190	0.205	0.207	0.168	0.251	0.225	0.258	0.245	0.290	0.321	0.321	0.380	0.329
	Qinghai	0.221	0.234	0.202	0.218	0.267	0.211	0.259	0.330	0.345	0.305	0.351	0.351	0.369	0.357	0.414
	Ningxia	0.222	0.188	0.247	0.247	0.253	0.239	0.286	0.310	0.368	0.386	0.362	0.384	0.408	0.416	0.386
	Xinjiang	0.191	0.239	0.224	0.237	0.235	0.269	0.331	0.297	0.318	0.374	0.403	0.335	0.354	0.367	0.354
	Mean	0.188	0.199	0.214	0.229	0.245	0.261	0.285	0.314	0.346	0.362	0.378	0.393	0.417	0.421	0.433
National		0.254	0.267	0.271	0.294	0.311	0.331	0.345	0.370	0.405	0.423	0.433	0.458	0.469	0.483	0.486

From Table 3, it can be seen that, from temporal trends: Firstly, the common prosperity level in China has shown an increasing tendency. Between 2006 and 2020, the mean level of common prosperity increased from 0.254 to 0.486. For a long time, there has been a concerted effort by China to incorporate the idea of "common prosperity" into its development programs^41^. Secondly, eastern China has seen greater levels of common prosperity than central China, while central China has experienced greater levels of common prosperity than western China. Eastern China, with its mature industrial chain, developed economy, and leading education and technology levels, has reached a state of relative affluence and was the first to start implementing the concept of getting rich first and getting rich later. Central and western China have comparatively slow economic growth, and their primary goal is still "affluence," and their affluence is not nearly as high as that of eastern China^42^. Finally, regional disparities in the degree of common prosperity are substantial among Chinese provinces from 2006 to 2020. In 2006, Beijing had the highest degree of common prosperity (0.472), following Zhejiang (0.406) and Shanghai (0.400). In comparison, Gansu (0.125), Yunnan (0.138), and Guizhou (0.139) had the lowest levels. In 2020, Beijing still had the highest level of common prosperity with 0.769, followed by Shanghai (0.667) and Zhejiang (0.587), while Gansu (0.329), Xinjiang (0.354), and Guizhou (0.360) had the lowest levels. This indicates that the common prosperity level is relatively high in economically developed provinces and relatively low in economically backward provinces. Compared with the economically backward provinces, the economically developed provinces have a higher level of affluence, sound public infrastructure of all kinds, a better sharing system for residents, and more attention to environmental protection and other aspects of construction^43^.

**Figure 2:**
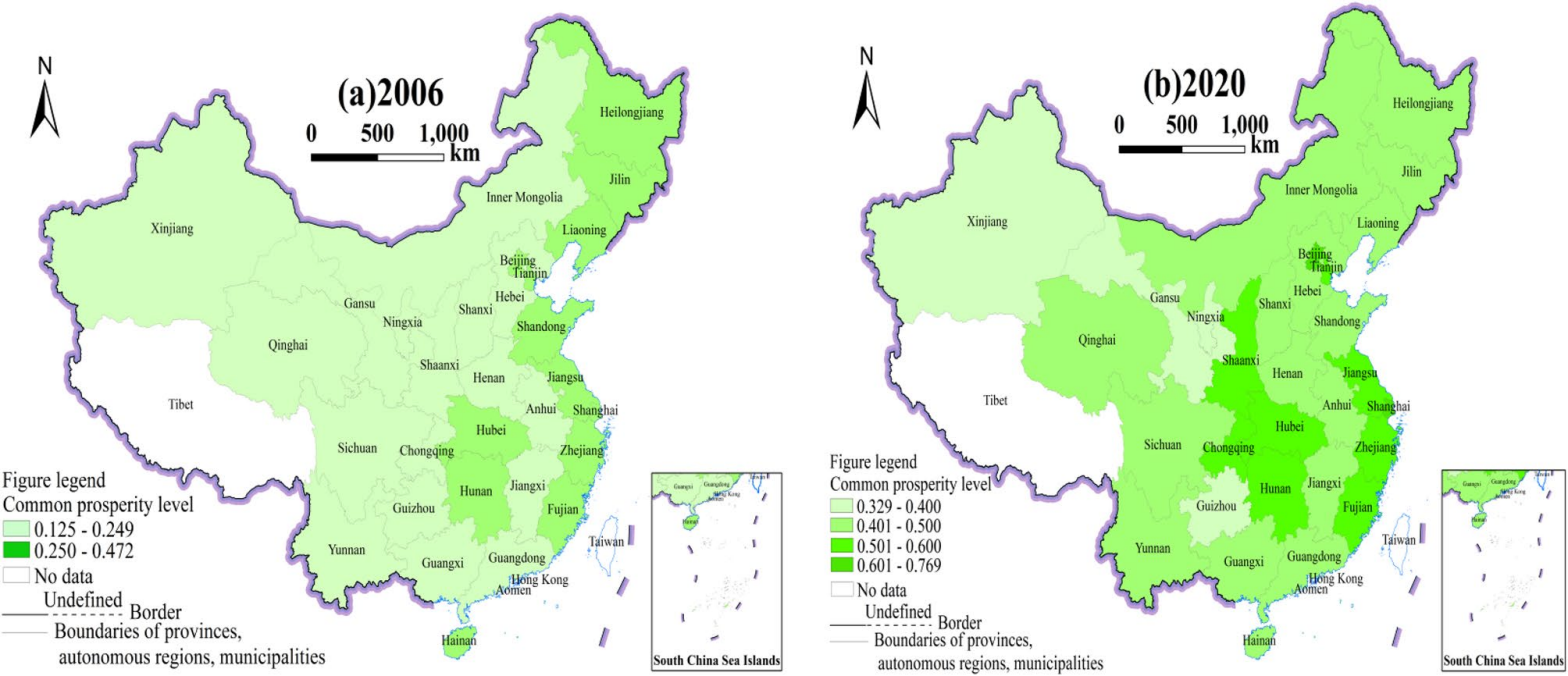
Provinces' common prosperity levels in 2006 and 2020. *Note* This figure is based on the standard map production of the National Base Map Service website of the Ministry of Natural Resources of China (http://bzdt.ch.mnr.gov.cn), with the map approval number of GS (2020) 4637, and the base map has not been modified. The software version used for map production is ArcGIS 10.8. URL link: https://www.esri.com/en-us/home.

To show more clearly the level changes and differences, the map of common prosperity levels in 30 Chinese provinces in 2006 and 2020 is plotted separately using equally spaced values, with darker colors indicating higher levels of common prosperity (see Fig. 2).

It can be observed that, in terms of spatial trends: In 2006, most provinces with higher levels of common prosperity in China were located in the developed eastern coastal and northeastern areas. The provinces in the western area generally had lower levels of common prosperity. The eastern coastal region is strategically located for easy access to the outside world, thriving international trade, and high levels of economic development^44^. The northeast area has been well-developed in terms of industrial industry since early on, ahead of the rest of China, and has correspondingly better economic development^45^. Hence, the common prosperity level in these two regional provinces is relatively high. Relatively speaking, the western provinces, which are inland, are not easily accessible and are also late in development^46^. In 2020, the general level of common prosperity increased throughout China's provinces, and the gap between regions narrowed significantly, with the central area developing more rapidly. This shows that the degree of common prosperity in China has been increasing after more than a decade of development, with provinces in the lagging regions catching up. Among them, the central area has continued to absorb and take a page out of the eastern area's book for success, so the level of common prosperity has increased faster^47^.

### The intensity of carbon emissions evaluation

The intensity of CO_2_ emissions in 30 Chinese provinces is calculated by applying Eq. (6). Table 4 displays the results.

**Table 4:** The intensity of carbon emissions in China from 2006 to 2020.

Region	Province	2006	2007	2008	2009	2010	2011	2012	2013	2014	2015	2016	2017	2018	2019	2020
Eastern China	Beijing	1.516	1.330	1.198	1.124	0.981	0.794	0.734	0.602	0.578	0.520	0.441	0.397	0.377	0.321	0.324
	Tianjin	2.967	2.683	2.089	2.006	1.997	1.787	1.578	1.452	1.287	1.207	1.055	1.008	1.031	1.386	1.415
	Hebei	5.414	4.981	4.406	4.366	3.970	3.735	3.494	3.268	3.004	3.098	2.884	2.702	2.617	2.697	2.652
	Liaoning	5.671	5.087	4.342	4.039	3.647	3.235	2.999	2.617	2.489	2.401	3.136	3.065	3.015	3.345	3.532
	Shanghai	2.161	1.878	1.760	1.638	1.571	1.445	1.355	1.311	1.104	1.039	0.926	0.869	0.801	0.709	0.710
	Jiangsu	2.417	2.180	1.858	1.746	1.622	1.577	1.462	1.354	1.235	1.188	1.120	1.000	0.919	0.873	0.855
	Zhejiang	2.149	2.012	1.794	1.741	1.551	1.408	1.274	1.175	1.093	1.036	0.932	0.894	0.810	0.747	0.740
	Fujian	1.930	1.785	1.593	1.669	1.540	1.475	1.305	1.143	1.187	1.058	0.895	0.845	0.841	0.756	0.786
	Shandong	3.734	3.450	3.051	2.899	2.774	2.523	2.409	2.115	2.104	2.183	2.117	2.038	1.923	2.122	2.123
	Guangdong	1.607	1.460	1.310	1.317	1.267	1.181	1.083	0.980	0.907	0.846	0.788	0.743	0.707	0.632	0.629
	Hainan	2.341	3.586	3.104	2.998	2.623	2.531	2.330	1.929	1.927	1.973	1.750	1.554	1.525	1.429	1.404
	Mean	2.901	2.767	2.409	2.322	2.140	1.972	1.820	1.631	1.538	1.504	1.459	1.374	1.324	1.365	1.379
Central China	Shanxi	12.911	10.891	8.618	8.487	7.282	6.573	6.367	6.240	6.343	7.280	7.047	6.240	6.170	6.412	6.551
	Jilin	4.311	3.681	3.422	3.091	2.896	2.717	2.377	2.086	1.954	1.635	1.535	1.507	1.547	2.052	1.997
	Heilongjiang	4.273	4.018	3.640	3.681	3.306	2.922	2.812	2.495	2.432	2.218	2.196	2.119	2.112	2.666	2.731
	Anhui	3.450	3.198	3.040	2.943	2.540	2.201	2.045	1.975	1.880	1.785	1.606	1.501	1.405	1.145	1.131
	Jiangxi	2.652	2.408	2.036	1.941	1.831	1.631	1.482	1.424	1.329	1.301	1.191	1.123	1.068	0.969	0.961
	Henan	3.895	3.552	3.053	2.886	2.633	2.488	2.118	1.918	1.785	1.590	1.435	1.275	1.185	0.970	0.930
	Hubei	3.557	3.202	2.588	2.429	2.262	2.093	1.848	1.429	1.308	1.148	1.032	0.972	0.910	0.830	0.913
	Hunan	2.983	2.814	2.273	2.115	1.828	1.663	1.455	1.262	1.116	1.039	0.973	0.913	0.869	0.794	0.764
	Mean	4.754	4.220	3.584	3.447	3.072	2.786	2.563	2.353	2.268	2.249	2.127	1.956	1.908	1.980	1.997
Western China	Inner Mongolia	10.194	6.595	5.932	5.634	5.181	5.257	4.939	4.526	4.415	4.365	4.340	5.140	5.489	6.117	6.689
	Guangxi	2.271	2.151	1.815	1.821	1.797	1.804	1.782	1.589	1.459	1.278	1.220	1.277	1.223	1.240	1.261
	Chongqing	2.978	2.708	2.287	2.182	1.986	1.790	1.550	1.195	1.147	0.912	0.833	0.784	0.753	0.655	0.632
	Sichuan	2.915	2.687	2.364	2.369	2.011	1.658	1.521	1.405	1.346	1.092	0.981	0.856	0.761	0.713	0.693
	Guizhou	8.125	7.120	5.905	5.888	5.045	4.503	4.100	3.604	3.036	2.680	2.504	2.190	1.844	1.668	1.554
	Yunnan	4.874	4.256	3.671	3.677	3.315	2.780	2.490	2.138	1.772	1.509	1.374	1.319	1.343	1.076	4.931
	Shaanxi	4.631	4.167	3.640	3.541	3.385	3.028	3.010	2.847	2.748	2.671	2.526	2.307	2.022	2.081	2.117
	Gansu	6.062	5.690	4.943	4.563	4.177	3.961	3.623	3.341	3.115	3.033	2.750	2.678	2.537	2.426	2.399
	Qinghai	4.874	4.657	3.989	3.826	3.057	2.921	3.072	3.005	2.582	2.272	2.487	2.334	2.093	1.997	1.936
	Ningxia	11.113	9.752	8.304	8.124	7.700	8.240	7.949	7.675	7.323	7.170	6.531	7.382	7.636	8.186	8.981
	Xinjiang	5.756	5.416	5.050	5.765	5.080	4.943	5.029	5.101	5.166	5.314	5.356	5.047	4.689	4.477	4.668
	Mean	5.800	5.018	4.354	4.308	3.885	3.717	3.551	3.311	3.101	2.936	2.809	2.847	2.763	2.785	3.260
National		4.458	3.980	3.436	3.350	3.028	2.829	2.653	2.440	2.306	2.228	2.132	2.069	2.007	2.050	2.234

From Table 4, it can be found that in terms of time trends: Firstly, the Chinese carbon emission intensity has generally been trending downward. Specifically, the annual average intensity of China's carbon emissions decreased from 4.458 in 2006 to 2.234 in 2020. This indicates that China's efforts to reduce carbon emissions have advanced significantly. China has actively undertaken the task of CO_2_ dioxide emission reduction, promulgated various environmentally friendly policies, and vigorously carried out afforestation and reforestation initiatives, which have gradually reduced the intensity of CO_2_ emissions^48^. Secondly, the three main regions' CO_2_ emission intensity likewise exhibits a decline in tendency between 2006 and 2020. Still, western China continues to have the greatest carbon emission intensity, following central China, while eastern China has the smallest. Western China has a weak economic base, and its industrial development is rough, which leads to high carbon emissions. In addition, its smaller population, complex terrain, and variable climate make technological innovation very difficult, with few green and low-carbon environmental industries. Hence, it has the highest intensity of CO_2_ emissions^49^. Central China has been introducing advanced technological resources and various talents from developed provinces in eastern China and gradually optimizing its industrial and energy structures. So its carbon emission intensity is lower^50^. After a dramatic improvement in their material standard of living, the people of eastern China have begun to give more attention to the content of their lives and have increased their environmental standards. The local government is working to create an environmentally friendly and sustainable economy, forcing technological innovation to help reduce carbon emissions, coupled with the talent and technology base accumulated in the early stages. So, the intensity of carbon emissions is the smallest^51^.

**Figure 3:**
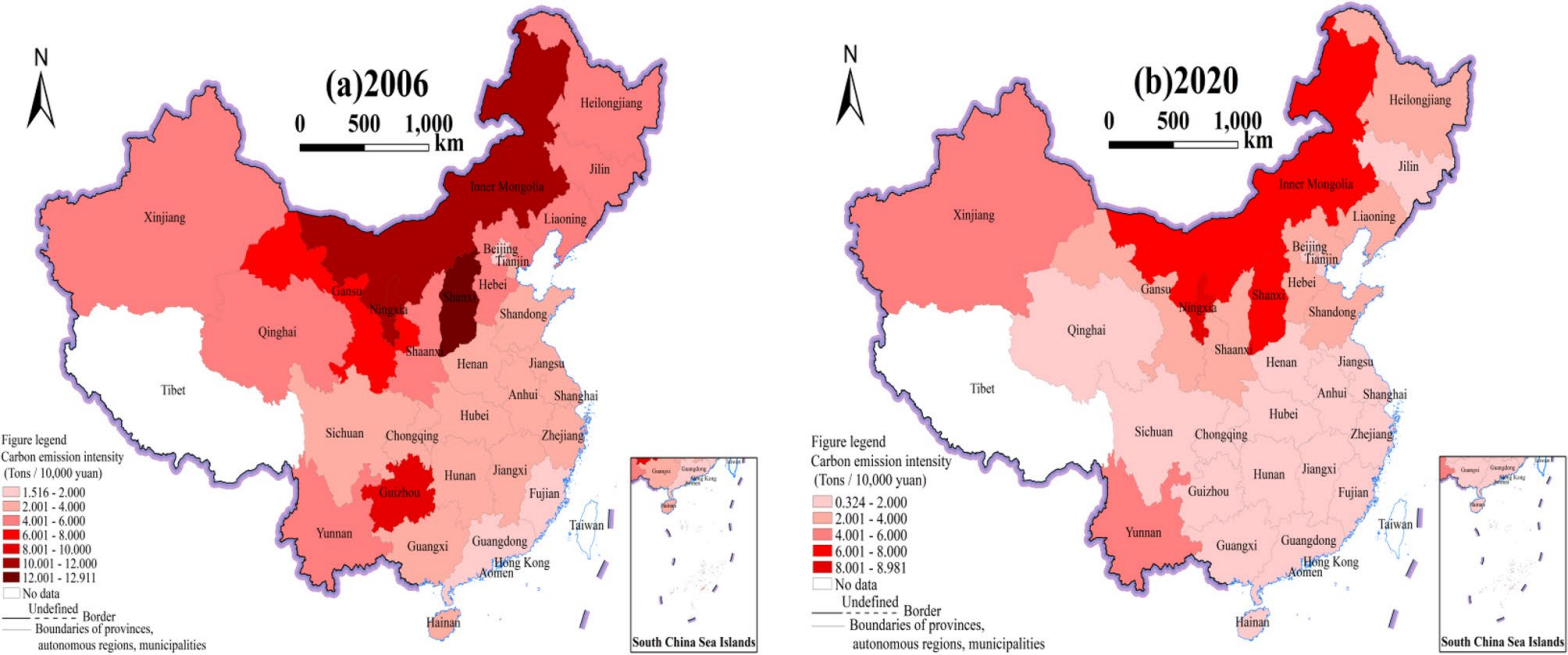
The intensity of carbon emissions by province in 2006 and 2020. *Note* This figure is based on the standard map production of the National Base Map Service website of the Ministry of Natural Resources of China (http://bzdt.ch.mnr.gov.cn), with the map approval number of GS (2020) 4637, and the base map has not been modified. The software version used for map production is ArcGIS 10.8. URL link: https://www.esri.com/en-us/home.

To show more clearly the intensity changes and differences, the map of the intensity of CO_2_ emissions in 30 Chinese provinces in 2006 and 2020 is also plotted separately using equally spaced values, with darker colors representing higher carbon emission intensity (see Fig. 3).

It can be observed that, in terms of spatial trends: In 2006, in general, compared to the southern and southeast coastal areas, the northern area had a greater intensity of carbon emissions, which was related to the industrial structure of each area. Most provinces in the northern area are dominated by heavy industrial industries with high fossil energy consumption, producing large amounts of CO_2_^52^. Compared with 2006, China's CO_2_ emission intensity decreased across the board by the year 2020, with more significant decreases in the northeast and western areas. This implies that the efforts made by local governments in China to conserve energy and reduce emissions are gradually bearing fruit. Environmental pollution and carbon emissions in northeast China have been taken seriously by the government. Environmental protection has been continuously implemented in the strategy to revitalize northeast China. Backward industries have been eliminated for industrial upgrading, which results in a continual reduction in the intensity of carbon emissions^53^. China proposed to develop conservation when implementing the western development strategy. As a result, the intensity of carbon emissions there has decreased significantly^54^. However, there were still certain provinces, nevertheless, where the intensity of CO_2_ emissions had decreased less, such as Inner Mongolia and Shanxi. Inner Mongolia's energy structure is relatively homogeneous, causing high carbon emissions. Together with the low emission reduction capacity, it results in a high intensity of CO_2_ emissions^55^. Shanxi has abundant coal resources and has formed a variety of coal-related industries, making it difficult to reduce carbon emission intensity^56^.

## Analysis of the effect of the common prosperity level on carbon emission intensity

### Spatial correlation test

Firstly, the global Moran index tests the spatial correlation of common prosperity level and carbon emission intensity. Table 5 displays the results. Table 5 shows that the global Moran indexes of China's common prosperity level and the intensity of carbon emissions in 2006–2020 are substantially positive at the 1% level for all three matrices, demonstrating a strong spatial association. However, the comparison shows that the global Moran index of common prosperity level under all three matrices is greater than 0.1, while the intensity of carbon emissions is greater than 0.1 only under the adjacency matrix (*W*_1_). Because the larger the Moran index, the stronger the agglomeration's performance. Therefore, it is more appropriate to analyze the influence of the common prosperity level on the intensity of CO_2_ emissions under the adjacency matrix (*W*_1_)^57^.

**Table 5:** Global Moran index.

Year	Common prosperity level			Carbon emission intensity		
	*W*_1_	*W*_2_	*W*_3_	*W*_1_	*W*_2_	*W*_3_
2006	0.363***	0.129***	0.165***	0.492***	0.105***	0.109***
2007	0.480***	0.156***	0.238***	0.417***	0.081***	0.136***
2008	0.436***	0.165***	0.224***	0.444***	0.095***	0.133***
2009	0.402***	0.145***	0.218***	0.426***	0.099***	0.128***
2010	0.389***	0.150***	0.205***	0.428***	0.093***	0.117***
2011	0.386***	0.135***	0.209***	0.417***	0.091***	0.111***
2012	0.421***	0.146***	0.208***	0.427***	0.097***	0.111***
2013	0.382***	0.109***	0.188***	0.416***	0.092***	0.106***
2014	0.481***	0.143***	0.166***	0.428***	0.095***	0.102***
2015	0.298***	0.115***	0.180***	0.389***	0.084***	0.098***
2016	0.296***	0.121***	0.188***	0.409***	0.087***	0.104***
2017	0.452***	0.115***	0.193***	0.429***	0.089***	0.099***
2018	0.455***	0.108***	0.188***	0.437***	0.091***	0.096***
2019	0.411***	0.112***	0.194***	0.459***	0.098***	0.097***
2020	0.310***	0.065***	0.155***	0.394***	0.081***	0.078***

The symbols *, **, and ***, respectively, represent significant at 10, 5, and 1% levels. The same is true in all of the tables below.

**Figure 4:**
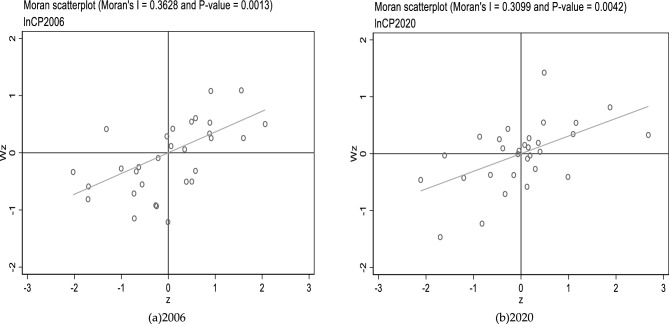
2006 and 2020 local Moran scatter plots of common prosperity.

**Figure 5:**
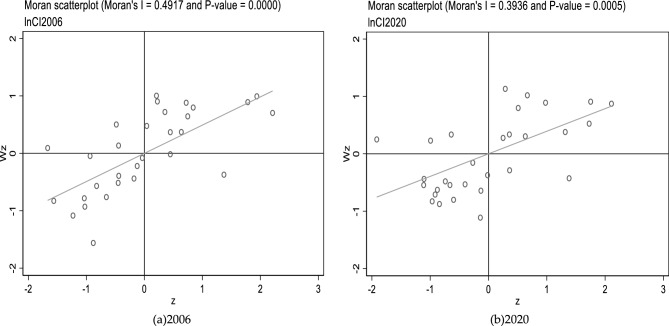
2006 and 2020 local Moran scatter plots of the intensity of carbon emissions.

Based on the adjacency matrix (*W*_1_), local Moran scatter plots are drawn by Stata software to investigate the spatial agglomeration status of the common prosperity level and the intensity of carbon emissions in every province. Figures 4 and 5 display the results.

The geographical agglomeration status of the common prosperity level in China's provinces is shown in Fig. 4 (the hollow circles indicate provinces). As can be seen in Fig. 4a, most Chinese provinces were in the first and third quadrants, i.e., in high-high and low-low agglomeration, and a few provinces were in low–high as well as high-low agglomerations in 2006. From Fig. 4b, the general spatial agglomeration state of each province's common prosperity level in 2020 did not show significant changes compared to 2006.

The geographical agglomeration status of the intensity of carbon emissions in China's provinces is shown in Fig. 5 (the hollow circles indicate provinces). From Fig. 5a, it can be found that most Chinese provinces were also in the first and third quadrants in 2006, and a few provinces were in low–high as well as high-low agglomerations. As can be seen in Fig. 5b, in 2020, the spatial agglomeration state of CO_2_ emission intensity in each province was likewise not significantly changed compared to 2006.

In conclusion, from the local spatial tests, Chinese provinces' common prosperity level and carbon emission intensity during 2006–2020 continue to show significant spatial aggregation. Most are in quadrants one and three with significant positive spatial correlation.

### Spatial effects measurement model selection

To test again whether the data fit the spatial econometric model and to select an appropriate spatial econometric model, model tests are conducted for the common prosperity level and the intensity of carbon emissions. Table 6 displays the results. Firstly, all LM (Lagrange Multiplier) tests passed significantly at the 1% level, meaning their suitability for spatial measurement studies. Secondly, the LR (Likelihood Ratio) results all passed significantly at the 1% level, showing that the SDM (Spatial Durbin Model) model does not degenerate into the SAR (Spatial Autoregressive Model) model or the SEM (Spatial Error Model) model, so the SDM model is the most suitable. Finally, the initial hypothesis is rejected at the 1% level using the Hausman test. Therefore, it should be analyzed under fixed effects. Based on the above, adopt the fixed effects SDM model to investigate the influence of common prosperity on the intensity of carbon emissions^58^.

**Table 6:** Model selection test.

Method		Test statistics	*P*
LM (Lagrange Multiplier)	LM-error	1332.248	0.000***
	Robust LM-error	1540.924	0.000***
	LM-lag	555.850	0.000***
	Robust LM-lag	37.307	0.000***
LR (Likelihood Ratio)	SDM-SAR	116.430	0.000***
	SDM-SEM	118.060	0.000***
Hausman		45.710	0.000***

### Spatial measurement results

The spatial lag term is calculated under the adjacency matrix (*W*_1_). Table 7 displays the results.

**Table 7:** SDM model regression results.

Variables	Coefficient	Standard error	Z	P	Confidence interval
ln*CP*	− 0.951	0.143	− 6.64	0.000***	[− 1.231581,− 0.6704377]
ln*IS*	0.880	0.110	8.01	0.000***	[0.6641835,1.09486]
ln*UR*	0.154	0.177	0.87	0.383	[− 0.1927761,0.5016753]
ln*TI*	− 0.225	0.019	− 12.06	0.000***	[− 0.2612222,− 0.1881839]
ln*OU*	0.128	0.031	4.16	0.000***	[0.067503,0.187712]
ln*FD*	0.157	0.083	1.89	0.058*	[− 0.0056162,0.3200786]
W*ln*CP*	− 0.778	0.299	− 2.60	0.009***	[− 1.364281,− 0.1909311]
W*ln*IS*	0.979	0.234	4.19	0.000***	[0.5207205,1.436867]
W*ln*UR*	1.916	0.349	5.48	0.000***	[1.23108,2.600847]
W*ln*TI*	0.024	0.031	0.61	0.545	[− 0.0540444,0.102375]
W*ln*OU*	− 0.234	0.065	− 3.60	0.000***	[− 0.3606056,− 0.1065764]
W*ln*FD*	− 0.060	0.146	− 0.41	0.684	[− 0.3461675,0.2271513]
Spatial rho	0.361	0.057	6.33	0.000***	[0.2489286,0.4724144]
sigma2_e	0.109	0.007	14.79	0.000***	[0.0938304,0.1225083]
R^2^	0.582	0.582	0.582	0.582	0.582
Obs	450	450	450	450	450

The spatial lag term (spatial rho) coefficient of whether there is a spatial impact of the intensity of CO_2_ emissions in 30 provinces is 0.361, as shown in Table 7, and it is significant at the 1% level. This shows a significant spatial aggregation effect of carbon emission intensity in Chinese provinces. The positive spatial spillover influence of the intensity of CO_2_ emissions is noticeable and significantly influenced by various factors in neighboring regions. The estimated coefficients of ln*CP*, lnIS, ln*TI*, ln*OU*, and ln*FD* are significant, at least at the 10% level, with common prosperity (ln*CP*) and technological innovation (ln*TI*) negatively related to carbon emission intensity. Industrial structure (ln*IS*), opening to the outside world (ln*OU*), and financial development (ln*FD*) are positively related to the intensity of carbon emissions. At the 1% level, the estimated coefficients of W*ln*CP*, W*ln*IS*, W*ln*UR*, and W*ln*OU* are significant, and the regional spatial spillover impact is noticeable. Among them, common prosperity (W*ln*CP*) and opening to the outside world (W*ln*OU*) have a negative relationship with the intensity of carbon emissions in neighboring regions. Industrial structure (W*ln*IS*) and urbanization (W*ln*UR*) have a positive relationship with the intensity of CO_2_ emissions in neighboring regions.

The SDM model's calculated coefficients cannot indicate the marginal influence of the explanatory factors on the explained variables. Therefore, the direct, spatial spillover, and total effect of variables such as common prosperity level on carbon emission intensity are further analyzed^59^. Table 8 displays the results.

**Table 8:** Decomposition results of the effect.

Explanatory variables	Direct effect	Spatial spillover effect	Total effect
ln*CP*	− 1.057*** (− 6.66)	− 1.643*** (− 3.38)	− 2.700*** (− 4.65)
ln*IS*	1.003*** (9.03)	1.888*** (5.08)	2.891*** (6.82)
ln*UR*	0.360 (1.98)	2.896*** (5.75)	3.256*** (5.50)
ln*TI*	− 0.231*** (− 11.81)	− 0.079 (− 1.40)	− 0.309*** (− 4.72)
ln*OU*	0.108*** (3.53)	− 0.284*** (− 3.01)	− 0.176* (− 1.65)
ln*FD*	0.156** (1.96)	0.004 (0.02)	0.160 (0.68)

z-values are in parentheses.

From Table 8, the regression coefficient of the direct effect of common prosperity level (ln*CP*) on CO_2_ emission intensity (ln*CI*) under the adjacency matrix (*W*_*1*_) is − 1.057 and is significant at the 1% level. It implies that the rise in the common prosperity level can significantly suppress the local region's intensity of CO_2_ emissions. This is because China pursues high-quality common prosperity, which is expressed as development, sharing, and sustainability. The term “development” refers to the economy's green development. In its early years, China consumed a large amount of fossil energy due to its blind pursuit of GDP growth, which led to a dramatic increase in CO_2_ emissions. Nowadays, China has recognized the necessity of improving environmental and ecological preservation, energy savings, and carbon reduction. It has begun to continuously develop a green and low-carbon circular economy, explore a sustainable development path, and use the funds generated by economic development to feed back into the cause of energy conservation and carbon reduction^60^. “Sharing” denotes the sharing of public social goods, and the people's per capita carbon emissions will be reduced through a shared lifestyle^61^. “Sustainability” includes technological innovation and ecological protection. First of all, for technological innovation, on the one hand, improving the technology level can improve energy use efficiency. On the other hand, the government has introduced policies to force technological innovation to help reduce carbon emissions, and CO_2_ dioxide emissions may be efficiently lowered by using cutting-edge technologies in environmental protection^62^. Secondly, regarding ecological protection, the Chinese government has vigorously promoted afforestation campaigns and established eco-forest demonstration areas. These initiatives have significantly increased the vegetation cover and helped reduce CO_2_ emission intensity^63^.

The regression coefficient of the spatial spillover effect of the common prosperity level (ln*CP*) on the intensity of carbon emissions (ln*CI*) is − 1.643 and passes the significance test at the 1% level. It suggests that increasing the common prosperity level can also suppress the intensity of carbon emissions in neighboring areas. This is because neighboring provinces have convenient exchanges, and relevant carbon reduction technologies and talents will flow to neighboring provinces. In addition, provinces with a low level of common prosperity will look to neighboring provinces that have higher levels. Learning from the experience of common prosperity construction, introducing technology and talents, and promoting their common prosperity level to improve continuously while curbing the intensity of CO_2_ emissions^64^. The regression coefficient for the total effect is − 2.700 and passes the significance test at the 1% level. Overall, common prosperity has a dampening influence on the intensity of CO_2_ emissions. This indicates that as the common prosperity level in China continues to increase, its effects on suppressing carbon emission intensity will become more apparent.

### Mediation effect results

According to Section “The influence mechanism of common prosperity on carbon emission intensity”, common prosperity affects carbon emission intensity through three aspects: economic growth, reducing the income disparity, and sharing economy. Then, we discuss these three mechanisms separately. Table 9 displays the results.

**Table 9:** Results of the mediation effect.

Variable	ln*CI*	ln*EG*	ln*CI*	ln*ID*	ln*CI*	ln*SE*	ln*CI*
	(1)	(2)	(3)	(4)	(5)	(6)	(7)
ln*CP*	− 0.071*** (− 6.96)	0.018*** (1.40)	− 0.096*** (− 5.30)	− 0.023** (− 2.36)	− 0.124*** (− 1.26)	0.034*** (4.16)	− 0.134*** (− 5.15)
ln*EG*			− 0.014** (− 1.12)				
ln*ID*					− 0.053*** (− 3.59)		
ln*SE*							− 0.101** (− 5.74)
_cons	− 0.831* (− 5.43)	0.536** (4.74)	− 2.281* (− 11.88)	− 1.343*** (− 4.63)	− 0.289** (− 7.85)	0.391*** (6.12)	− 0.719*** (− 4.92)
Control variables	Yes						
Time fixed effect	Yes						
Province fixed effects	Yes						
R^2^	0.962	0.957	0.933	0.576	0.616	0.784	0.832

The symbols ***, **, and * denote statistical significance at the 1, 5, and 10% levels, respectively; the number contained in parentheses is the z-statistic.

#### Mediation effect of economic growth

Columns (1)–(3) of Table 9 show the estimated results of the mediation effect of ln*EG* in the effect of common prosperity on carbon emission intensity. Column (1) displays the effects of common prosperity on carbon emissions intensity. The regression coefficient of the effect of common prosperity level (ln*CP*) on carbon emissions intensity (ln*CI*) is − 0.071 and is significant at the 1% level. Once again, it is verified that the development of common prosperity can curb the intensity of carbon emissions. While column (2) indicates the influence of common prosperity on economic growth. The regression coefficient of the effect of common prosperity level (ln*CP*) on economic growth (ln*EG*) is 0.018 and is significant at the 1% level. This demonstrates that common prosperity significantly contributes to economic development. This is because shared prosperity helps create a more inclusive growth model that fosters social stability and overall prosperity. And in Column (3) displays the effects of economic growth on carbon emissions intensity. The regression coefficient of the effect of economic growth (ln*EG*) on carbon emissions intensity (ln*CI*) is − 0.014 and is significant at the 5% level. This suggests that economic development during the process of common prosperity will have a dampening effect on carbon emission intensity. This may be attributed to China's pursuit of high-quality common prosperity and the development of a green economy. Within the framework of common prosperity, the Chinese government has implemented various measures during the course of economic growth, including technological innovation, industrial upgrading, policy and regulatory changes, and shifts in the energy structure. These measures have, in turn, contributed to the reduction of carbon emission intensity.

#### Mediation effect of income disparity

Columns (1), (4), and (5) of Table 9 show the estimated results of the the mediation effect of ln*ID* in the effect of common prosperity on carbon emission intensity. According to the estimated outcomes in column (4), common prosperity acts as a disincentive to widening income disparities. The common prosperity strategy, through a series of policies and measures such as resource redistribution, societal support, and reduction of social inequality, helps mitigate the widening income gap in society. The coefficient of ln*ID* is negative, as seen in column (5), which suggests that reduced income disparities can significantly reduce carbon emission intensity. Narrowing the income gap can enhance public consensus on environmental protection, thereby assisting the government in formulating more effective low-carbon environmental policies and improving their implementation.

#### Mediation effect of sharing economy

The estimated outcomes of the mediating role of ln*SE* in the influence of common prosperity on carbon emission intensity are displayed in columns (1), (6), and (7). Column (6) demonstrates that common prosperity promotes sharing economy development. The common prosperity process strongly supports the healthy development of the sharing economy. It achieves this by increasing resource sharing, lowering participation barriers, promoting entrepreneurship, and fostering social acceptance, all of which collectively contribute to the robust growth of the sharing economy. And in Column (7) displays the effects of sharing economy (ln*SE*) on carbon emissions intensity. The regression coefficient of the effect of sharing economy (ln*SE*) on carbon emissions intensity (ln*CI*) is − 0.101 and is significant at the 5% level. This suggests that sharing economic development in the process of common prosperity will have a dampening effect on carbon emission intensity. The sharing economy is usually centred on resource sharing and optimal utilization, which can effectively reduce the waste of resources and energy. In addition, the sharing economy encourages people to use resources in a more rational and environmentally friendly manner, which can promote a low-carbon lifestyle.

## Conclusion and discussion

### Research findings

According to the context of a low-carbon economy, sustainable development, and China's pursuit of common prosperity, according to the panel data of 30 Chinese provinces from 2006 to 2020, this study measures the level of common prosperity and the intensity of carbon emissions in China. Then, the SDM model is applied to analyze the influence of the common prosperity level on the intensity of carbon emissions. According to the results of the data analysis, draw four main research conclusions.

First, China's common prosperity level is gradually increasing. The common prosperity level in eastern China is higher than that in central China and western China. The Chinese government has been implementing the concept of "common prosperity" in its development and actively promoting the construction of common prosperity^65^. Eastern China is economically developed and has reached a state of relative affluence with its leading education and technology levels^66^. It is the first to start implementing the concept of "getting rich first and getting rich later." In comparison, the economic growth of central and western China is behind, and the main goal is to achieve “affluence”^67^.

Second, the intensity of China's carbon emissions is trending downward. Western China has the greatest carbon emission intensity, following central China, while eastern China has the smallest. China has actively undertaken the task of carbon emission reduction, promulgated various energy-saving and emission-reduction policies, and vigorously carried out afforestation and reforestation initiatives, which have gradually reduced carbon emission intensity^68^. Western China has a weak economic base, and its industrial development is mainly rough, which leads to high carbon emissions^69^. So its carbon emission intensity is the highest. Central China has been introducing advanced technological resources and various talents from developed provinces in eastern China and gradually optimizing its industrial and energy structures^70^. So its carbon emission intensity is lower. Eastern China has developed a low-carbon economy, forcing technical advancement to help reduce carbon emissions, coupled with the talent and technology base accumulated in the early stages^71^. Therefore, the intensity of carbon emissions is the lowest.

Third, both the common prosperity level and carbon emission intensity in China exhibit positive spatial autocorrelation at a significant level of 1%. The level of common prosperity in neighboring provinces affects each other. The intensity of carbon emissions in neighboring provinces also affects each other. This is because neighboring provinces have convenient exchanges, and technologies, funds, and talents will flow to neighboring provinces. A strong correlation exists between neighboring areas that affect each other^72^.

Fourth, the SDM model test shows that the increase in common prosperity level significantly inhibits the intensity of carbon emissions in both local and neighboring areas. Overall, common prosperity plays a suppressive role in the intensity of CO_2_ emissions. China pursues high-quality common prosperity, expressed as "development, sharing, and sustainability." The "development" indicates the green development of the economy. China has developed a green economy that is circular and low-carbon, explored a route towards sustainable growth, and used the funds generated by economic growth to feed back into the cause of lowering carbon emissions^73^. "Sharing" denotes the sharing of public social goods, and the people's per capita carbon emissions will be reduced through a shared lifestyle^74^. "Sustainability" includes technological innovation and ecological protection. Firstly, improving the technology level can improve energy efficiency. Secondly, regarding ecological protection, the Chinese government has vigorously promoted afforestation campaigns and established eco-forest demonstration areas. These initiatives have significantly increased the vegetation cover and helped reduce carbon emission intensity^75^. In addition, provinces with a low common prosperity level will look to neighboring provinces with a higher level. Learning from the experience of common prosperity construction, introducing technology and talents, and promoting their common prosperity level to improve continuously while curbing carbon emission intensity^76^.

Fifth, the mediating effects model suggests that the common prosperity process suppresses carbon emission intensity through high-quality economic development, the reduction of income disparity, and the promotion of the sharing economy.

## Research recommendations

The following three suggestions are made in light of the results above. Firstly, strengthen regional cooperation and gradually reduce regional disparities in the degree of common prosperity and carbon emission intensity. China's central and western regions must increase their common prosperity level further. Compared with eastern China, western and central China are less developed and have lower levels of common prosperity, emitting considerable amounts of carbon dioxide in the quest for economic progress. In the future, central and western China should learn from the development experience of eastern China, complement its strengths and weaknesses, and gradually narrow the gap with eastern China^77^. Secondly, promote the construction of common prosperity and raise the level of common prosperity to curb carbon emission intensity. Common prosperity can suppress the intensity of CO_2_ emissions in local and neighboring regions. Under the macro-planning of the central government, each area should realize common prosperity scientifically and reasonably according to its development, raise the level of common prosperity, and continuously suppress carbon emission intensity in the realization of common prosperity^78^. Thirdly, the mediating effects model suggests that the common prosperity process suppresses carbon emission intensity through high-quality economic development, the reduction of income disparity, and the promotion of the sharing economy. Therefore, in the pursuit of common prosperity, it becomes essential to further develop the green economy, consistently narrow income gaps, and vigorously promote the sharing economy.

### Theoretical contribution

This paper makes three significant contributions to the existing literature. First, research on carbon emissions is currently a popular subject in academia. Many academics have analyzed the factors influencing CO_2_ emissions. Meanwhile, as the economy has grown, China has put common prosperity in an important position. China's construction of common prosperity has far-reaching implications. However, little literature has focused on the influence of common prosperity on CO_2_ emissions^79^. The construction of common prosperity is a systematic project that includes harmonious relationships between humans and the environment and sustainable economic and social development. It is bound to impact carbon emissions in the process of realizing common prosperity. Thus, this study helps enrich the research content on carbon emission influencing factors to a certain extent. Second, this article constructs the common prosperity indicator system from three perspectives: sustainability, sharing, and development, which can reflect the level of common prosperity more comprehensively and accurately^80^. Third, China is now actively promoting the construction of common prosperity and the "double carbon goal." The analysis of the influence of common prosperity on the intensity of CO_2_ emissions could provide a reference for the scientific development of CO_2_ emission reduction strategies in the process of achieving common prosperity^81^. This is significant for promoting China's low-carbon transition and achieving sustainable development. Thirdly, the mediating effects model indicates that the common prosperity process suppresses carbon emission intensity through high-quality economic development, the reduction of income disparity, and the development of a sharing economy. Therefore, in the pursuit of common prosperity, it is essential to further foster the green economy, continually narrow income gaps, and vigorously promote the sharing economy.

## Research limitations and future prospects

The thesis still has some shortcomings and needs further improvement. Firstly, this article explores the influence of common prosperity on CO_2_ emission intensity at the provincial level. In the future, based on the premise that data are available, the effect of common prosperity on carbon emissions can be further explored from the city's perspective^82^. Secondly, due to a large amount of missing data for regions such as Taiwan, Macao, Tibet, and Hong Kong, software simulations can be used in the future to complete the data for these regions and start the analysis. Finally, the analysis method used in this paper is the SDM model. In the future, other research methods can be used to explore the impact of common prosperity on carbon emissions and obtain new findings^83,84^.

## Data Availability

The article's data is provided in a publicly accessible repository without DOIs. This research investigated publicly accessible datasets. This information was accessible through the following: [https://navi.cnki.net/knavi/yearbooks/YCXME/detail?uniplatform=NZKPT, accessed on 6 May 2023] [https://navi.cnki.net/knavi/yearbooks/YINFN/detail?uniplatform=NZKPT, accessed on 6 May 2023] [https://navi.cnki.net/knavi/yearbooks/YBVCX/detail?uniplatform=NZKPT, accessed on 6 May 2023].
